# Correlation Between Sinonasal Outcome Test-22 (SNOT-22) Scores and Patient Satisfaction With Nasal Breathing After Septoplasty With Inferior Turbinate Reduction

**DOI:** 10.7759/cureus.79500

**Published:** 2025-02-23

**Authors:** João Tavares Correia, Francisco Sousa, Isabel Silva-Carvalho, Luís Meireles, Mariline Santos

**Affiliations:** 1 Otolaryngology - Head and Neck Surgery, Unidade Local de Saúde de Santo António, Porto, PRT

**Keywords:** nasal breathing, nasal obstruction, nasal surgery, patient satisfaction, quality of life

## Abstract

Aim: The study aimed to correlate the variation in the Sinonasal Outcome Test-22 (SNOT-22) scores (preoperative vs. postoperative) with the Visual Analog Scale (VAS) satisfaction scores for nasal breathing three months after septoplasty with inferior turbinate reduction.

Materials and methods: Adult patients with nasal obstruction due to deviated nasal septum were assessed. Preoperative and three-month postoperative SNOT-22 results, mean ∆SNOT-22 values, and VAS satisfaction scores for nasal breathing were recorded and analyzed.

Results: A total of 26 patients were included in the study. Postoperative SNOT-22 scores significantly decreased at three months compared to preoperative scores (p=0.001), with a mean ∆SNOT-22 score of 23.38±23.39. The three-month postoperative VAS satisfaction score for nasal breathing was 8.54±1.75. Pearson correlation analysis revealed a significant correlation between SNOT-22 and VAS satisfaction scores three months postoperatively (r=0.482, p=0.017).

Conclusion: Patients undergoing septoplasty with inferior turbinate reduction for nasal obstruction exhibit high satisfaction levels with nasal breathing three months postoperatively. The VAS satisfaction scores correlate with improved SNOT-22 scores post-surgery.

## Introduction

Nasal obstruction, a prevalent symptom encountered by primary care physicians and otolaryngologists, can stem from various anatomical, physiological, and pathophysiological factors. Approximately one-third of the population experiences nasal obstruction and seeks medical intervention for symptom relief [1]. Anatomical causes of nasal obstruction encompass internal or external nasal valve stenosis/collapse, septal deviations, and inferior turbinate hypertrophy, among others. Septoplasty and inferior turbinate reduction are commonly performed surgical procedures to address nasal obstruction caused by septal deviation and turbinate hypertrophy, respectively [2,3].

The use of quality-of-life questionnaires such as patient-reported outcome measures (PROMs) plays a central role in assessing the subjective perception of the impact of a specific symptom or disease on patients’ daily lives [4]. The Sinonasal Outcome Test-22 (SNOT-22) is a questionnaire consisting of 22 questions, primarily used to assess the impact of chronic rhinosinusitis (CRS) on patients’ quality of life [5]. On the other hand, the Nasal Obstruction Symptom Evaluation (NOSE) is a valid, simple, and reproducible instrument that quantifies the impact attributable to nasal obstruction and can be used to evaluate the outcome of septoplasty [6,7]. A recent study suggests that SNOT-22 correlates well with NOSE and, therefore, can also be used to assess the outcome of septoplasty and/or inferior turbinate reduction surgery, regardless of the coexistence of CRS [8]. The Visual Analog Scale (VAS) is a widely used psychometric response scale for quantifying the impact of various diseases [9].

Therefore, our study aimed to investigate the correlation between the variation in SNOT-22 scores (preoperative vs. postoperative) and patients’ satisfaction with nasal breathing three months after surgery. The improvement in SNOT-22 scores (preoperative vs. postoperative) was also analyzed.

## Materials and methods

Adult patients (aged >18 years) experiencing nasal obstruction due to septal deviation and inferior turbinate hypertrophy scheduled for surgical intervention at Unidade Local de Saúde de Santo António between March and October of 2023 were consecutively enrolled in this prospective study.

Exclusion criteria comprised the following: (i) patients undergoing other concurrent surgical procedures (e.g., functional endoscopic sinus surgery, rhinoplasty, adenoidectomy, or uvulopalatoplasty); (ii) patients with a history of previous septal surgery; and (iii) failure to respond to either preoperative or postoperative questionnaires.

Patients completed the SNOT-22 questionnaire in person both preoperatively and three months postoperatively. Only the final scores were registered for analysis. The SNOT-22 scores range from 0 to 110, with higher scores indicating a greater impact of the nasal condition on quality of life. Additionally, satisfaction scores for nasal breathing were collected in person at three months post-surgery using a VAS ranging from 0 to 10, where “0” represents complete dissatisfaction and “10” indicates complete satisfaction with their current nasal breathing.

Categorical data were noted as percentages, while continuous data were noted as means and standard deviations. Skewness and kurtosis were used to assess normality. The differences between mean preoperative and postoperative SNOT-22 scores were assessed using a paired sample test. The correlation between ∆SNOT-22 and VAS scores for nasal breathing satisfaction three months after surgery was examined using the Pearson correlation test. Statistical analyses were conducted using IBM SPSS Statistics for Windows v29 (IBM Corp., Armonk, US), with a significance level set at p<0.05.

Septoplasty was performed using the Cottle technique and combined with inferior turbinate reduction via radiofrequency plasma ablation with the BONSS® system (BONSS Medical, Jiangsu, China). All surgeries were carried out by the same surgeon, a second-year ear, nose, and throat (ENT) resident. Internal silicone splints (Silastic®; BioPlexus, Ventura, US) were placed to stabilize the corrected septal position and reduce the risk of postoperative complications, including bleeding and hematoma. Nasal packing (PosiSepX®; Hemostasis LLC, St. Paul, US) was applied to ensure immediate hemostasis. All patients received the same postoperative care, with splints and nasal packing being consistent for every patient. Patients were instructed to perform nasal saline irrigation four times daily. Splints were removed, and nasal packing was aspirated seven days after surgery.

The study was approved by the Ethics Committee of Centro Hospitalar Universitário de Santo António and Instituto de Ciências Biomédicas Abel Salazar (CHUdSA/ICBAS) (approval no. 2021-234(186 DEFI/194-CE)) and adhered to the Declaration of Helsinki. Informed consent was obtained from all participants.

## Results

A total of 26 patients were enrolled in this study, consisting of 13 male and 13 female participants, with a mean age of 34.92±12.29 years (Table 1). The mean preoperative SNOT-22 score was 45.77±19.51, while the mean SNOT-22 score at three months postoperatively was 22.38±17.23, resulting in a mean ∆SNOT-22 score of 23.38±23.39 (Table 1). The mean satisfaction score on the VAS for nasal breathing at three months postoperatively was 8.54±1.75 (Table 1).

**Table 1:** Baseline characteristics and PROMs scores of the population PROMs: patient-reported outcome measures, SNOT-22: Sinonasal Outcome Test-22.

Baseline characteristics and PROMs scores	All patients (N=26)
Sex (male), n (%)	13 (50)
Age, mean±SD	34.92±12.29
Submitted to septoplasty and inferior turbinate reduction, n (%)	26 (100)
SNOT-22 score (preoperative), mean±SD	45.77±19.51
SNOT-22 score (three months postoperative), mean±SD	22.38±17.23
∆SNOT-22 score, mean±SD	23.38±23.39
VAS score (three months postoperative), mean±SD	8.54±1.75

Notably, the postoperative SNOT-22 score at three months was significantly lower than the preoperative score (p=0.003), as illustrated in Table 2.

**Table 2:** Preoperative vs. three months postoperative SNOT-22 scores The differences were assessed using a paired sample test. SNOTpre: preoperative SNOT-22 score; SNOT3m: three months postoperative SNOT-22 score; SNOT-22: Sinonasal Outcome Test-22.

	Mean	SD	Lower	Upper	One-sided p	Two-sided p
SNOTpre - SNOT3m	23.38	23.39	10.15	38.62	0.001	0.003

Additionally, the Pearson correlation test demonstrated a statistically significant correlation between the ∆SNOT-22 score and VAS satisfaction score for nasal breathing at three months postoperatively (r=0.482, p=0.017), as illustrated in Table 3.

**Table 3:** Statistic correlation between ∆SNOT-22 and VAS scores The correlation was assessed using the Pearson correlation test. *: Correlation is significant at the 0.05 level (2-tailed); SNOT-22: Sinonasal Outcome Test-22; VAS: Visual Analog Scale.

		VAS score
∆SNOT-22	Pearson Correlation	0.482*
	Sig. (2-tailed)	0.017
	N	26

## Discussion

The findings of this study offer valuable insights into the efficacy of septoplasty and inferior turbinate reduction in alleviating nasal obstruction symptoms and enhancing the overall quality of life for patients with deviated nasal septum and inferior turbinate hypertrophy. Nasal obstruction is a multifaceted issue stemming from various anatomical, physiological, and pathophysiological factors, affecting a significant portion of the population and prompting many to seek medical intervention [1-3].

Quality-of-life questionnaires like the SNOT-22 and NOSE are pivotal in evaluating the impact of nasal conditions on patients' daily lives [5-7]. The correlation between SNOT-22 and NOSE scores reinforces their utility in assessing treatment outcomes, as evidenced by their association with surgical interventions such as septoplasty and inferior turbinate reduction. Our study demonstrated a significant reduction in three-month postoperative SNOT-22 scores compared to preoperative scores, indicating substantial improvement in nasal symptoms following surgery. In the literature, a decrease of 8.9 points is considered the minimal clinically important difference (MCID) when the SNOT-22 is used in the context of surgical treatment for CRS [10]. It may be reasonable to apply a similar MCID for patients with a deviated septum and turbinate hypertrophy treated with septoplasty and inferior turbinate reduction. Notably, our study observed a mean difference exceeding 20 points, supporting the conclusion that the procedure significantly improved patients’ quality of life. This finding aligns with prior research, which has consistently demonstrated the efficacy of septoplasty and inferior turbinate reduction in relieving nasal obstruction. For example, a study by Stewart et al. reported that patients undergoing septoplasty experienced marked improvements in NOSE scores, high levels of satisfaction, and reduced reliance on medication [11].

Moreover, correlation analysis indicated a significant association between ∆SNOT-22 scores and patients' satisfaction with nasal breathing, as measured by VAS. This correlation underscores the importance of patient-reported outcomes in evaluating treatment efficacy and patient satisfaction. Indeed, research by Shukla et al. highlighted the effectiveness of VAS scores in evaluating treatment responses following septoplasty [12].

These findings have significant clinical implications for managing nasal obstruction, providing further evidence supporting septoplasty and inferior turbinate reduction as effective treatment options for patients with a deviated nasal septum and inferior turbinate hypertrophy. They also emphasize the importance of incorporating patient-reported outcome measures into clinical practice to comprehensively assess treatment outcomes and tailor interventions to individual patient needs.

In conclusion, our study underscores the positive impact of septoplasty and inferior turbinate reduction on nasal obstruction symptoms and patient satisfaction with nasal breathing. Notably, to the best of the authors' knowledge, this study represents the first attempt to correlate SNOT-22 scores with patient satisfaction regarding nasal breathing following septoplasty and inferior turbinate reduction.

While these results are promising, the study has some limitations. These include a relatively small sample size, an exclusive focus on specific patient demographics, a limited follow-up period, reliance on patient-reported outcome measures, and the absence of objective measures of nasal airflow or anatomical changes post-surgery. Furthermore, reporting the preoperative and postoperative differences in each subdomain of the SNOT-22 could have enhanced the study's value. Addressing these limitations in future research would improve the evidence base and deepen our understanding of surgical interventions for nasal obstruction.

## Conclusions

Patients experiencing nasal obstruction due to a deviated nasal septum and inferior turbinate reduction who undergo septoplasty with inferior turbinate reduction exhibit a high level of satisfaction with nasal breathing at three months postoperatively. Furthermore, the satisfaction scores on the VAS for nasal breathing at this time point correlates with the improvement observed in SNOT-22 scores following the surgical intervention.
